# Phaeoviruses discovered in kelp (Laminariales)

**DOI:** 10.1038/ismej.2017.130

**Published:** 2017-07-25

**Authors:** Dean A McKeown, Kim Stevens, Akira F Peters, Peter Bond, Glenn M Harper, Colin Brownlee, Murray T Brown, Declan C Schroeder

**Affiliations:** 1 Viral Ecology, Marine Biological, Association, Citadel Hill, Plymouth, UK; 2 School of Biological and Marine Sciences, University of Plymouth, Drake Circus, Plymouth, UK; 3 Bezhin Rosko, Santec, France; 4 Electron Microscopy Centre, University of Plymouth, Drake Circus, Plymouth, UK; 5 School of Ocean and Earth Sciences, University of Southampton, Southampton, UK; 6 School of Biological Sciences, University of Reading, Reading, UK

## Abstract

Phaeoviruses are latent double-stranded DNA viruses that insert their genomes into those of their brown algal (Phaeophyceae) hosts. So far these viruses are known only from members of the Ectocarpales, which are small and short-lived macroalgae. Here we report molecular and morphological evidence for a new *Phaeovirus* cluster, referred to as sub-group C, infecting kelps (Laminariales) of the genera *Laminaria* and *Saccharina*, which are ecologically and commercially important seaweeds. Epifluorescence and TEM observations indicate that the *Laminaria digitata* Virus (LdigV), the type species of sub-group C, targets the host nucleus for its genome replication, followed by gradual degradation of the chloroplast and assembly of virions in the cytoplasm of both vegetative and reproductive cells. This study is the first to describe phaeoviruses in kelp. In the field, these viruses infected two thirds of their host populations; however, their biological impact remains unknown.

Kelps (brown algae of the order Laminariales) are the largest marine photosynthetic organisms, engineering temperate rocky coastlines into complex habitats comparable to terrestrial forests, that support extensive marine ecosystems (Dayton, 1985). Ectocarpoids (order Ectocarpales) are small filamentous brown algae (Cock *et al.*, 2010) sharing habitat and close evolutionary relationships with kelps (Kawai *et al.*, 2015). Ectocarpoids are host to the only fully characterised seaweed viruses (genus *Phaeovirus*), which are comprised of nine virus species infecting seven ectocarpoid species. Phaeoviruses are eukaryotic algal viruses (family *Phycodnaviridae*) with large (150–350 kb), complex double-stranded DNA genomes (Schroeder, 2011), and are Nucleo-Cytoplasmic Large DNA Viruses alongside *Poxviridae*, *Asfarviridae*, *Iridoviridae*, *Ascoviridae*, and *Mimiviridae*. The well-studied type species of *Phaeovirus* sub-group A is *Ectocarpus siliculosus virus* 1 (EsV-1), which infects *Ectocarpus siliculosus* using a persistent strategy, integrating its genome into the genome of hosts infected during their short term as motile spores or gametes, that is, the only wall-less life-cycle stages (Maier *et al.*, 2002). Each host cell inherits the phaeoviral genome, but symptoms appear only in reproductive organs (sporangia or gametangia), which are reprogrammed to produce virus particles instead of host zoids (Müller, 1996). Phaeoviral diversity and host range are largely unknown (Park *et al.*, 2011; Schroeder, 2011). For example, Stevens *et al.* (2014) provided evidence that members of the *Phaeovirus* sub-group B (mainly viruses that infect the ectocapoid *Feldmannia*) evolved from sub-group A, through genome reduction and accompanying loss of DNA proofreading capability. This has led to *Phaeovirus* increasing its host range and changing from a *K-* to an *r-* strategist (Stevens *et al.*, 2014).

There are approximately 13.5 thousand described seaweed species (Guiry, 2012), with 13% belonging to the brown algal class Phaeophyceae. Seaweeds are possibly host to a vast, unexplored diversity of viruses. There have been microscopic observations of virus-like particles (VLPs) in some seaweeds other than Ectocarpales, but none are described in any greater detail (Schroeder, 2011). We have examined three ecologically and commercially important European kelp species, *Laminaria digitata*, *L. hyperborea* and *Saccharina latissima*, targeting the phaeovirus-encoded major capsid protein (MCP) gene using a standard PCR methodology (Stevens *et al.*, 2014). Samples were taken on both sides of the English Channel (Supplementary Figure S1); they included 34 field sporophyte tissue samples collected from epiphyte-free meristematic zones, mixtures of gametophytes isolated from 82 fertile field sporophytes, and 28 clonal gametophyte cultures isolated from gametophyte mixtures that were MCP positive in PCR. In total, 64.7% of sporophytes and 23.2% of gametophyte mixes were phaeoviral MCP positive (Supplementary Table S1).

Phylogenetic analysis of 28 sequences of MCP fragments from kelps (160 bp in length; Figure 1), obtained by Sanger sequencing of cloned PCR products showed that the viral sequences from Laminariales (sporophyte MCPs: KY063706-KY063723 and gametophyte MCPs: HG003317-HG003355, LdigPH10-30 m: KY316507) formed a cluster distinct from all known phaeoviruses, which we name sub-group C (Figure 1; posterior probability 0.91). Sub-group C appears to share common ancestry with sub-group A (posterior probability 0.93) and sub-group B (posterior probability 0.87). Similar phaeoviral variants in sub-group C were found in *Laminaria* and *Saccharina*, suggesting a host range including multiple genera. The gametophytes LdigPH10-18 and SlatPH10-7 each had 2 different viral MCP sequences, which suggests multiple infection in a single host individual (Figure 1). Given that a combined MCP-DNA polymerase phylogeny of ectocarpoid phaeoviruses showed similar sub-group distinctions (Stevens *et al.*, 2014; Schroeder, 2015), it is unknown if sub-group C diverged at the same time as sub-group B, that is, during or after the speciation of the Ectocarpales, or during or after the divergence of Ectocarpales and Laminariales 90.5 Ma (Kawai *et al.*, 2015). If algal viruses are ancient, then ancestral phaeoviruses may have expanded their host range into all brown algal orders. Many brown algal groups need to be screened for viruses, followed by phylogenetic analyses of any new viral sequences. This would allow the common descent and lateral transfer of brown algal viruses to be disentangled.

To further describe the sub-group C kelp phaeoviruses, we focused on the *L. digitata* strain LdigPH10-30 m (Supplementary Table S1), a male gametophyte culture that produced an array of consistent phaeoviral infection-like symptoms (Figures 2a–n), alongside normal growth and gametogenesis (Figure 2a). Gametangia formed preferentially on short side branches (Figure 2a), with one to several spermatozoids developing in each (~5 μm in diameter, arrowhead Figure 2a). The gametes were ejected through a mucilaginous cap, leaving empty translucent gametangia (white arrow, Figure 2a). Female *L. digitata* gametophyte strains (LdigPH10-31f and LdigPH10-22f) showed similar phaeoviral infection symptoms (Supplementary Figure S2). Healthy gametophyte cells have a large nucleus that can be visualised through DAPI staining and epifluorescence microscopy (discrete and localised blue fluorescence, white arrowheads Figures 2b and c); these are often closely associated with chloroplasts (large irregular red auto-fluorescent structures, Figures 2a–c) distributed around the cell periphery (Figure 2e). Heavily DAPI stained cells were associated with many opaque and not translucent cells (Figures 2b–d). It has been previously reported that similar cells in Ectocarpales were a result of viral infection and that the phaeovirus DNA genomes could be detected through DAPI staining (Müller *et al.*, 1990).

Transmission electron microscopy (TEM) of the *L. digitata* strain LdigPH10-30 m suggests that LdigV-1, similar to phaeovirus infections in Ectocarpales, targets the nucleus resulting in the eventual degeneration (Figures 2f and g) as the cytoplasm fills with long tubular structures (arrows; Figures 2h, i and k), followed by the development of virus-like particles (VLPs) (Figures 2f–l). Simultaneously, the chloroplasts detached from the cell periphery and lost their internal structure and pigmentation (Figure 2f). After nuclear and chloroplast degeneration, more fully formed VLPs were visible in the cytoplasm (Figures 2j–l). VLPs were 80–150 nm in diameter, with a 60–100 nm granular core (Figures 2l and n). The VLPs appeared round to hexagonal and may have icosahedral capsids, as known in other phaeoviruses. Mature VLPs were observed in ultrafiltered gametophyte culture medium (Figures 2m and n) showing a structure similar to intracellular VLPs. Our observations in kelp compare well with the characteristics of EsV-1 in *Ectocarpus* as described by Müller *et al.* (1990). However, unlike the ectocarpoid phaeoviruses, the infection in kelp appears to be common in vegetative cells (Figures 2d and e) and we do not know yet how the virions are released. Examination of the sorus of field sporophytes did not reveal any abnormal structures, suggesting that kelp viruses, unlike those in Ectocarpoids, may only be expressed in the gametophytes.

Natural reservoirs of gametophytes stabilise kelp populations by allowing new sporophyte recruitment (Steneck *et al.*, 2002) following natural or anthropogenic deforestation (Dayton, 1985; Dayton *et al.*, 1999; Smale *et al.*, 2013). Sea surface temperature increases of 1.4–5.8 °C over the next century (Cox *et al.*, 2000; Houghton *et al.*, 2001) may cause local extinctions of European kelps (Raybaud *et al.*, 2013). In ectocarpoids, phaeoviral symptoms are temperature sensitive (Müller *et al.*, 1998), but it is unknown how phaeoviruses will affect the biology and ecology of their ectocarpoid and kelp hosts in future climate change scenarios.

Seaweeds are exploitated for human consumption, livestock feed (MacArtain *et al.*, 2007; Evans and Critchley, 2014), unique polysaccharides with many industrial applications, pharmaceuticals (Kraan, 2012; Schiel and Foster, 2015), bioremediation, and biofuel (Kraan, 2013). Knowledge of kelp phaeoviruses may be required to meet challenges to seaweed aquaculture (Cottier-Cook *et al.*, 2016), especially since phaeoviruses are transmitted through the germline and could have unexpected effects in cultivation conditions. Though the effects of viruses on kelps remain to be studied in detail, if phaeoviruses commonly occur in kelps they may be transmitted in the host genome, and could alter host reproduction. The discovery of phaeoviruses in kelps highlights the need to further explore the diversity, biology, and ecology of brown algal viruses.

## Figures and Tables

**Figure 1:**
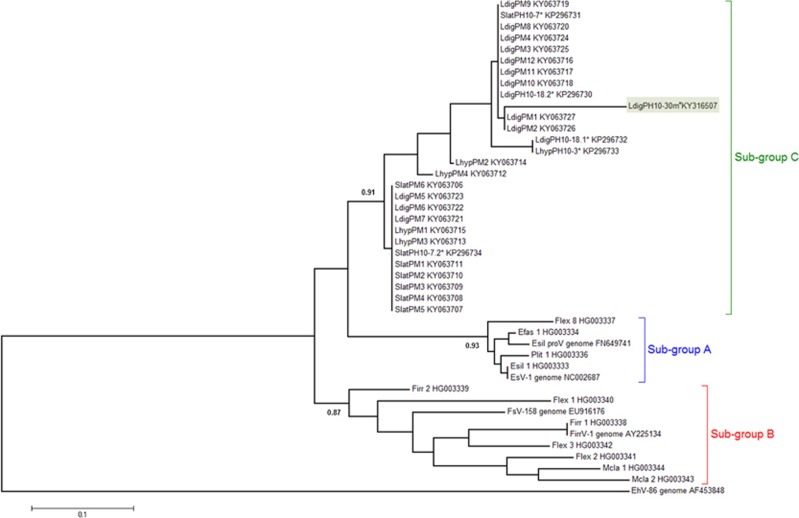
Phylogenetic analysis of sub-group C phaeoviral MCP from Laminariales and Ectocarpales phaeoviral sub-groups A and B. The Coccolithovirus EhV-86 (Schroeder *et al.*, 2002) was used as an outgroup. Topology based on maximum likelihood and decimals are Bayesian posterior probabilities for each sub-group. * denotes sequence variants from gametophyte isolates. Accession numbers are given for each sequence. Scale bar denotes number of nucleotide substitutions per site. Highlighted in green is the strain used for microscopy observations.

**Figure 2:**
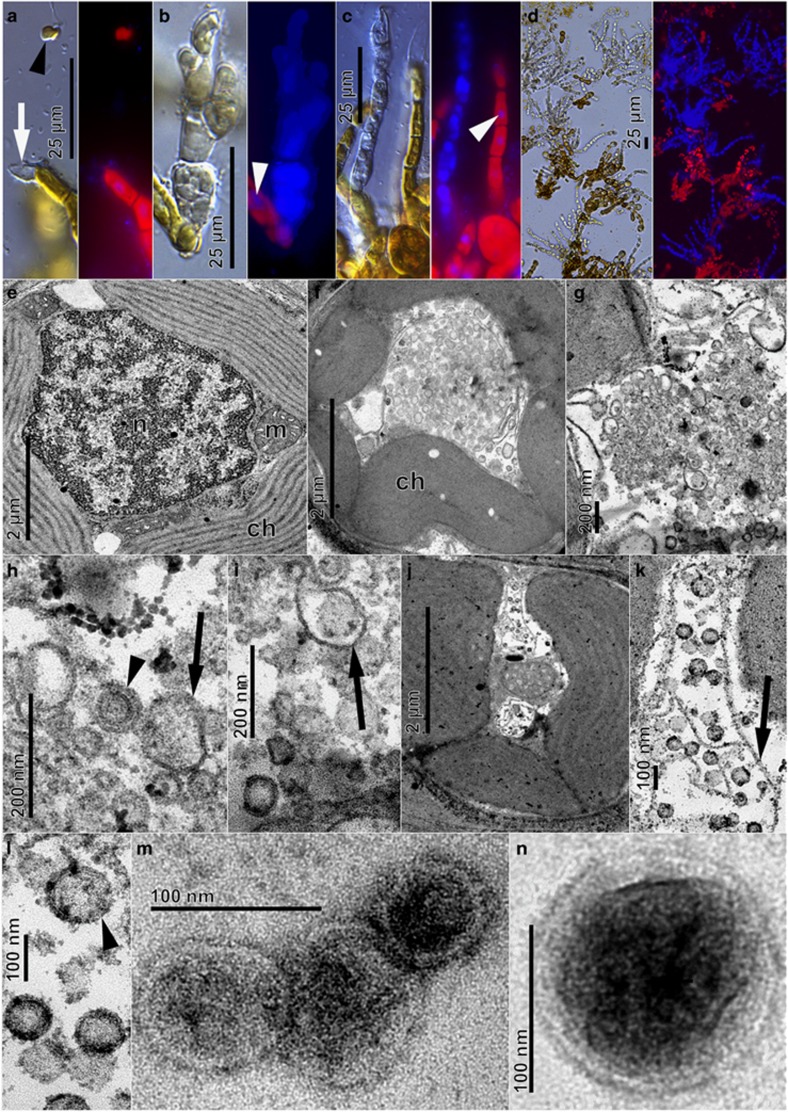
Light and epifluorescence (**a**–**d**, DAPI stained) and transmission electron (**e**–**n**) micrographs of *Laminaria digitata* gametophyte strain LdigPH10-30 m. (**a**) Spermatozoid (arrowhead) released from antheridium (white arrow), (**b**,**c**). Deformed opaque structures with high DAPI blue fluorescence in contrast to normal nuclei (white arrowheads). (**d**) High prevalence of DAPI-fluorescent filaments. (**e**) Cross-section of healthy vegetative cell showing chloroplast (ch), nucleus (n), and mitochondria (m). (**f**–**l**) VLP formation in vegetative gametophyte cells. Chloroplasts detached from cell periphery, loss of internal structure, appearance of tubular structures (arrows) and various stages of VLP assembly (arrowheads). (**m**,**n**) VLPs isolated from extracellular medium and visualised by negative staining.
