# Supplementary material for: Phaeoviruses discovered in kelp (Laminariales)
Source: ISME J. 2017 Jul 25;11(12):2869–73. doi: 10.1038/ismej.2017.130 (PMC5702736; doi:10.1038/ismej.2017.130)
Supplement: Supplementary Information [file ismej2017130x1.docx]

**
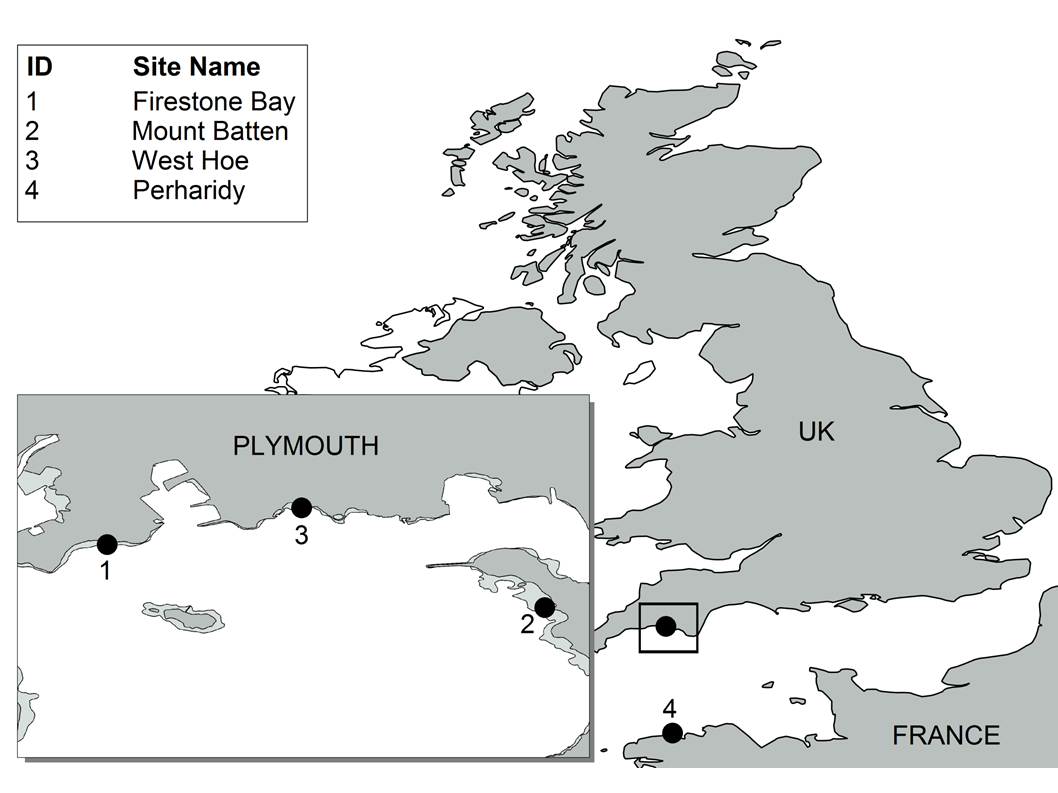
**

**Supplementary Fig. S1.** Collection sites of cultured gametophytes and wild sporophytes.

**
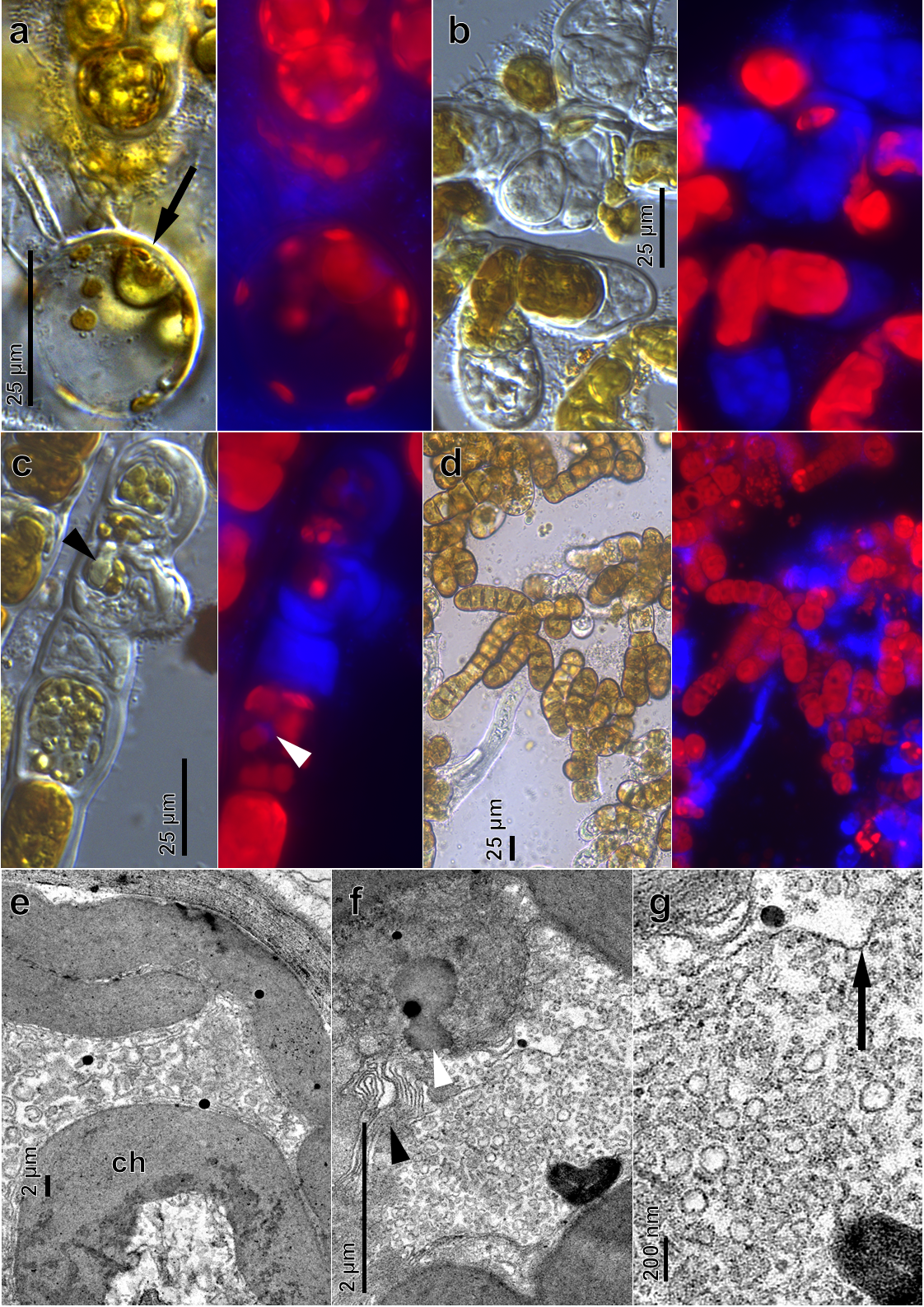
**

**Supplementary Fig. S2.** Light and epifluorescence (**a-d**, DAPI stained) and transmission electron (**e-g**) micrographs of female *Laminaria* *digitata* gametophyte strains LdigPH10-31f (**b-g**) and LdigPH10-22f (**a**). (**a**) Normal female gamete (arrow), (**b**) Deformed opaque structures with high DAPI blue fluorescence, (**c**) deformed structure with partially degraded chloroplasts (arrowhead) and opaque, DAPI-fluorescent material in contrast to healthy nuclei (white arrowhead), and (**d**) prevalent putative virus-filled structures in female gametophyte culture. Cross-sections of vegetative cells showing (**e**) degraded chloroplasts (ch) which have detached from cell periphery and lost internal structure, (**f**) VLP formation in vegetative gametophyte cells with putative degraded chloroplast (arrowhead) and nucleus (white arrowhead), and appearance of tubular structures (arrow) and early stages of VLP assembly in cytoplasm.

**Supplementary Table S1.** Wild sporophytes and cultured kelp gametophytes screened for phaeoviral MCP.

| Species | Sample details, e.g. life history generation | Sample or strain designation | Phaeoviral MCP PCR result | Site* | Date |
| --- | --- | --- | --- | --- | --- |
| *Laminaria digitata* | Sporophyte | LdigPM13 | - | 1 | 21.3.15 |
| *Laminaria digitata* | Sporophyte | LdigPM1 | + | 1 | 21.3.15 |
| *Laminaria digitata* | Sporophyte | LdigPM2 | + | 1 | 21.3.15 |
| *Laminaria digitata* | Sporophyte | LdigPM3 | + | 1 | 21.3.15 |
| *Laminaria digitata* | Sporophyte  Sporophyte | LdigPM4 | + | 1 | 21.3.15 |
| *Saccharina latissima* | Sporophyte | SlatPM1 | + | 1 | 22.3.15 |
| *Saccharina latissima* | Sporophyte | SlatPM7 | - | 1 | 22.3.15 |
| *Saccharina latissima* | Sporophyte | SlatPM2 | + | 1 | 22.3.15 |
| *Saccharina latissima* | Sporophyte | SlatPM8 | - | 1 | 22.3.15 |
| *Saccharina latissima* | Sporophyte | SlatPM9 | - | 1 | 22.3.15 |
| *Laminaria digitata* | Sporophyte | LdigPM5 | + | 2 | 21.3.15 |
| *Laminaria digitata* | Sporophyte | LdigPM6 | + | 2 | 21.3.15 |
| *Laminaria digitata* | Sporophyte | LdigPM14 | - | 2 | 21.3.15 |
| *Laminaria digitata* | Sporophyte | LdigPM7 | + | 2 | 21.3.15 |
| *Laminaria digitata* | Sporophyte | LdigPM15 | - | 2 | 21.3.15 |
| *Saccharina latissima* | Sporophyte | SlatPM3 | + | 2 | 22.3.15 |
| *Saccharina latissima* | Sporophyte | SlatPM4 | + | 2 | 22.3.15 |
| *Saccharina latissima* | Sporophyte | SlatPM10 | - | 2 | 22.3.15 |
| *Saccharina latissima* | Sporophyte | SlatPM11 | - | 2 | 22.3.15 |
| *Saccharina latissima* | Sporophyte | SlatPM12 | - | 2 | 22.3.15 |
| *Laminaria digitata* | Sporophyte | LdigPM8 | + | 3 | 21.3.15 |
| *Laminaria digitata* | Sporophyte | LdigPM9 | + | 3 | 21.3.15 |
| *Laminaria digitata* | Sporophyte | LdigPM10 | + | 3 | 21.3.15 |
| *Laminaria digitata* | Sporophyte | LdigPM11 | + | 3 | 21.3.15 |
| *Laminaria digitata* | Sporophyte | LdigPM12 | + | 3 | 21.3.15 |
| *Laminaria hyperborea* | Sporophyte | LhypPM1 | + | 3 | 21.3.15 |
| *Laminaria hyperborea* | Sporophyte | LhypPM2 | + | 3 | 21.3.15 |
| *Laminaria hyperborea* | Sporophyte | LhypPM3 | + | 3 | 21.3.15 |
| *Laminaria hyperborea* | Sporophyte | LhypPM4 | + | 3 | 21.3.15 |
| *Saccharina latissima* | Sporophyte | SlatPM5 | + | 3 | 22.3.15 |
| *Saccharina latissima* | Sporophyte | SlatPM6 | + | 3 | 22.3.15 |
| *Saccharina latissima* | Sporophyte | SlatPM13 | - | 3 | 22.3.15 |
| *Saccharina latissima* | Sporophyte | SlatPM14 | - | 3 | 22.3.15 |
| *Saccharina latissima* | Sporophyte | SlatPM15 | - | 3 | 22.3.15 |
| *Laminaria digitata* | Gametophyte mixtures | LdigPH10-1 to 10-48 | 13/48 + | 4 | 11.8.10 |
| *Laminaria digitata* | Gametophyte clone | LdigPH10-6m | - | 4 | 11.8.10 |
| *Laminaria digitata* | Gametophyte clone | LdigPH10-10f | + | 4 | 11.8.10 |
| *Laminaria digitata* | Gametophyte clone | LdigPH10-10m | + | 4 | 11.8.10 |
| *Laminaria digitata* | Gametophyte clone | LdigPH10-11f | + | 4 | 11.8.10 |
| *Laminaria digitata* | Gametophyte clone | LdigPH10-11f | - | 4 | 11.8.10 |
| *Laminaria digitata* | Gametophyte clone | LdigPH10-18f | - | 4 | 11.8.10 |
| *Laminaria digitata* | Gametophyte clone | LdigPH10-18m | + | 4 | 11.8.10 |
| *Laminaria digitata* | Gametophyte clone | LdigPH10-21f | + | 4 | 11.8.10 |
| *Laminaria digitata* | Gametophyte clone | LdigPH10-21m | - | 4 | 11.8.10 |
| *Laminaria digitata* | Gametophyte clone | LdigPH10-22f | + | 4 | 11.8.10 |
| *Laminaria digitata* | Gametophyte clone | LdigPH10-22m | + | 4 | 11.8.10 |
| *Laminaria digitata* | Gametophyte clone | LdigPH10-24f | - | 4 | 11.8.10 |
| *Laminaria digitata* | Gametophyte clone | LdigPH10-24m | - | 4 | 11.8.10 |
| *Laminaria digitata* | Gametophyte clone | LdigPH10-25m | - | 4 | 11.8.10 |
| *Laminaria digitata* | Gametophyte clone | LdigPH10-30f | - | 4 | 11.8.10 |
| *Laminaria digitata* | Gametophyte clone | LdigPH10-30m | + | 4 | 11.8.10 |
| *Laminaria digitata* | Gametophyte clone | LdigPH10-31f | + | 4 | 11.8.10 |
| *Laminaria digitata* | Gametophyte clone | LdigPH10-31m | - | 4 | 11.8.10 |
| *Laminaria digitata* | Gametophyte clone | LdigPH10-33f | + | 4 | 11.8.10 |
| *Laminaria digitata* | Gametophyte clone | LdigPH10-33m | + | 4 | 11.8.10 |
| *Laminaria digitata* | Gametophyte clone | LdigPH10-43m | + | 4 | 11.8.10 |
| *Laminaria digitata* | Gametophyte clone | LdigPH10-44f | + | 4 | 11.8.10 |
| *Laminaria digitata* | Gametophyte clone | LdigPH10-44m | + | 4 | 11.8.10 |
| *Saccharina latissima* | Gametophyte mixtures | SlatPH10-1 to 24 | 5/24 + | 4 | 7.11./23.12.10 |
| *Saccharina latissima* | Gametophyte clone | SlatPH10-2m | - | 4 | 7.11.10 |
| *Saccharina latissima* | Gametophyte clone | SlatPH10-7f | - | 4 | 7.11.10 |
| *Saccharina latissima* | Gametophyte clone | SlatPH10-7m | + | 4 | 7.11.10 |
| *Saccharina latissima* | Gametophyte clone | SlatPH10-10m | + | 4 | 7.11.10 |
| *Saccharina latissima* | Gametophyte clone | SlatPH10-20f | - | 4 | 23.12.10 |
| *Laminaria hyperborea* | Gametophyte mixtures | LhypPH10-1 to 10-10 | 1/10 + | 4 | 24.12.10/20.1.11 |

*: As identified in Supplementary Fig. S1
